# HElmet therapy Assessment in infants with Deformed Skulls (HEADS): protocol for a randomised controlled trial

**DOI:** 10.1186/1745-6215-13-108

**Published:** 2012-07-09

**Authors:** Renske M van Wijk, Magda M Boere-Boonekamp, Catharina GM Groothuis-Oudshoorn, Leo A van Vlimmeren, Maarten J IJzerman

**Affiliations:** 1 Department of Health Technology and Services Research, Institute for Governance Studies, University of Twente, P.O. Box 217, 7500 AE, Enschede, The Netherlands; 2 Department of Rehabilitation, Pediatric Physical Therapy, Radboud University Nijmegen Medical Centre, P.O. Box 9101, 6500 HB, Nijmegen, The Netherlands; 3 Scientific Institute for Quality of Health Care, Radboud University Nijmegen Medical Centre, P.O. Box 9101, 6500 HB, Nijmegen, The Netherlands

## Abstract

**Background:**

In The Netherlands, helmet therapy is a commonly used treatment in infants with skull deformation (deformational plagiocephaly or deformational brachycephaly). However, evidence of the effectiveness of this treatment remains lacking. The HEADS study (HElmet therapy Assessment in Deformed Skulls) aims to determine the effects and costs of helmet therapy compared to no helmet therapy in infants with moderate to severe skull deformation.

**Methods/design:**

Pragmatic randomised controlled trial (RCT) nested in a cohort study. The cohort study included infants with a positional preference and/or skull deformation at two to four months (first assessment). At 5 months of age, all children were assessed again and infants meeting the criteria for helmet therapy were asked to participate in the RCT. Participants were randomly allocated to either helmet therapy or no helmet therapy. Parents of eligible infants that do not agree with enrolment in the RCT were invited to stay enrolled for follow up in a non-randomisedrandomised controlled trial (nRCT); they were then free to make the decision to start helmet therapy or not. Follow-up assessments took place at 8, 12 and 24 months of age. The main outcome will be head shape at 24 months that is measured using plagiocephalometry. Secondary outcomes will be satisfaction of parents and professionals with the appearance of the child, parental concerns about the future, anxiety level and satisfaction with the treatment, motor development and quality of life of the infant. Finally, compliance and costs will also be determined.

**Discussion:**

HEADS will be the first study presenting data from an RCT on the effectiveness of helmet therapy. Outcomes will be important for affected children and their parents, health care professionals and future treatment policies. Our findings are likely to influence the reimbursement policies of health insurance companies.

Besides these health outcomes, we will be able to address several methodological questions, *e.g.* do participants in an RCT represent the eligible target population and do outcomes of the RCT differ from outcomes found in the nRCT?

**Trial registration:**

ISRCTN18473161.

## Background

Infants have malleable and fast-growing cranial bones, and are therefore at risk of developing skull deformation if their head often remains in the same position. When a child turns its head toward one side most of the time, this is defined as positional preference [1]. Skull deformation due to such prolonged external forces (non-synostotic) must be distinguished from skull malformation due to premature fusion of the cranial sutures (synostotic) [2]. Deformational brachycephaly refers to a symmetric occipital flattening of the skull that is sometimes accompanied by temporal bossing or an occipital lift [3]. The term deformational plagiocephaly is used to describe a unilateral occipital flattening of the skull. More severe cases often present with ear misalignment and facial asymmetry [2,4].

Skull deformation is generally considered a purely cosmetic disorder. Yet parents worry that the deformation might be permanent and might influence the child’s attractiveness with the risk of, for example, being teased [5]. Some studies suggest long-term developmental delays due to skull deformity, but no causal relationships have been found [5-7].

The prevalence of skull deformation can be up to 21.5% in infants younger than 6 months, but decreases within the first years of life [8-10]. A low parental level of education, ethnicity, male gender, primiparity, prematurity, birth factors, delayed (motor) development, low activity level and several positioning and dietary factors have been reported as risk factors, while placing a child in the prone position when awake appears to be a protective factor [1,11-17].

Prevention or treatment of positional preference and skull deformation include parental counselling, counter-positioning and physical therapy [10,18]. Children with persisting severe skull deformation at the age of 5 to 6 months are commonly treated using orthotic devices (redression helmets or headbands) [4,19]. In The Netherlands, a redression helmet costs about €1,200 and is reimbursed by health insurance companies as well as the accompanying visits to the (paediatric) physician. However, until now, no randomised controlled trials (RCTs) have been performed to study the effectiveness of this therapy [20,21]. The few non-randomised studies tend to show positive results, but have several limitations. To start with it is unknown whether the reported differences in effectiveness are clinically relevant. Furthermore, follow-up in these studies was short-term (either directly after treatment or just a few months afterwards), there was a lack of blinding or information about blinding and often no validated outcome measures were used. Finally, data about complications were not collected in a structural way in these studies [2,4,20,22,23]. Although the known complications of helmet therapy are mild and do not seem to occur often, the treatment burdens both parents and their young children [22]. Next to the lack of scientific evidence, experience shows differences in beliefs and referral policies of health care professionals regarding helmet therapy. Some advocate the use of helmets to treat skull deformation, while others are reluctant to prescribe this intensive treatment for a cosmetic condition without knowing its effectiveness [21,24]. This makes parents very uncertain when they have to decide whether to start helmet therapy or not.

Both helmet therapy and no helmet therapy (allowing natural recovery) are standard approaches in The Netherlands. To compare the effectiveness of these two approaches a pragmatic RCT study design is required [25]. Pragmatic trials are designed to find out the effectiveness of a treatment in routine, everyday practice and thereby have a high external validity [26,27]. A high external validity can be achieved by recruiting a broad study population that is representative of the target population, studying interventions that approach a real world delivery of care, applying blinding to neither participants nor specialists and selecting a wide range of outcome measures [28,29].

Since the condition of interest changes over time and the decision-making is time-dependent, the RCT needs to be nested in a cohort study [30-32]. The decision to start helmet therapy is usually taken at 5 to 6 months of age. Recruitment at that stage is complicated as the children tend to be scattered among various institutes if their parents prefer helmet therapy or are outside the health care system if their parents choose not to start helmet therapy. As the cohort study recruits children at risk of disease progression before helmet therapy can be prescribed, we tackle this problem and we are also able to predict the number of children that ultimately will be eligible for helmet therapy and identify prognostic factors.

Additionally, nesting the RCT in a prospective cohort study makes it possible to present information on the representativeness of the RCT population, by comparing this population with non-participants [33,34]. Furthermore, outcomes of the randomised trial can be compared with the parallel non-randomised trial that employs the same types of intervention.

The main goal of the Helmet Therapy Assessment in Deformed Skulls (HEADS) study is to investigate the effects and costs of six months of helmet therapy compared to no helmet therapy in children with moderate to severe skull deformation. This article describes how this study is designed and reports the recruitment scheme so far. We provide a description of the statistical analysis plan to be used after data collection is completed and conclude with general recommendations on study design.

## Methods/design

### Study design

**Figure 1:**
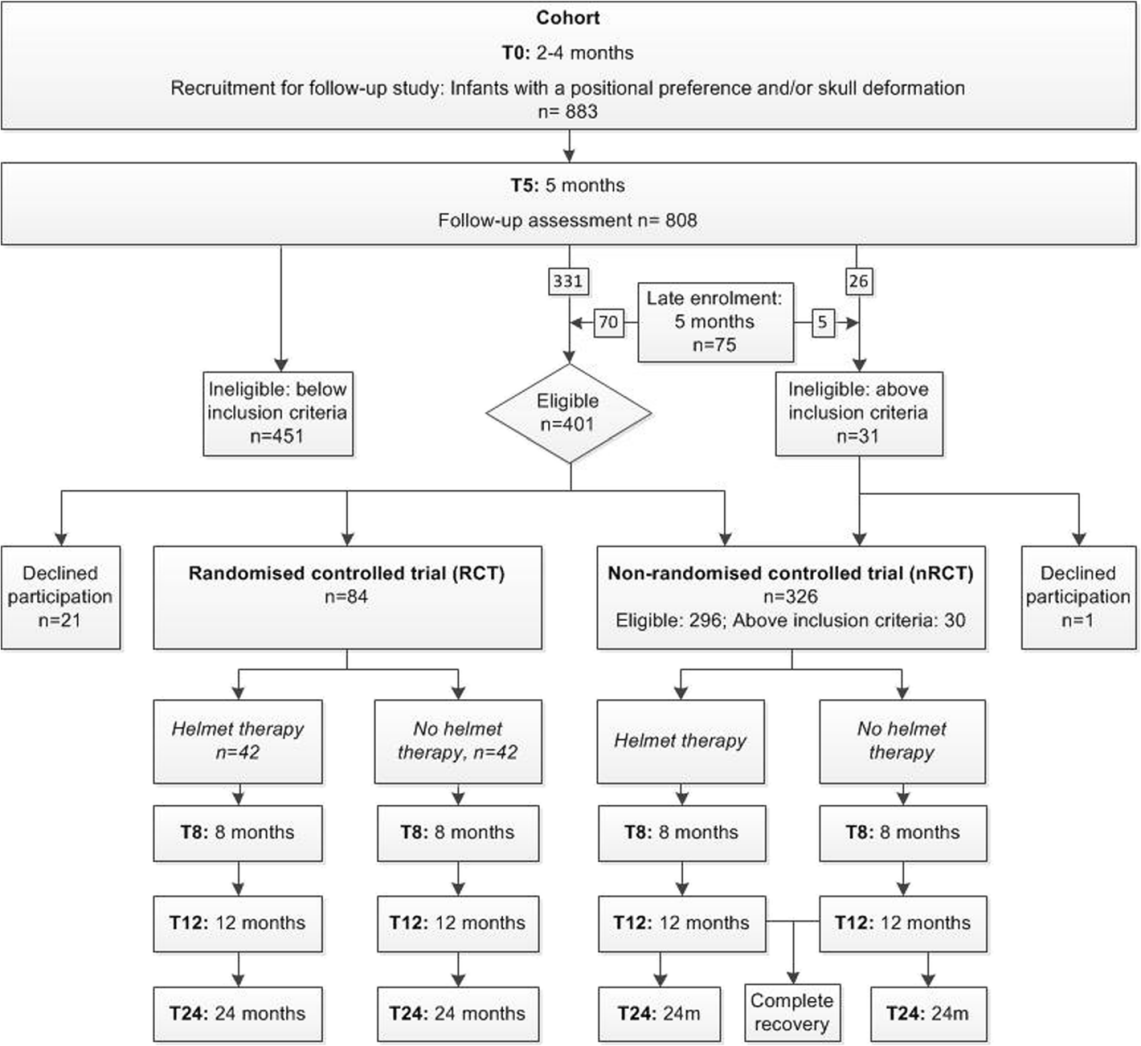
**Flow chart of participants in HEADS.** Provisional data.

The HEADS study is a two-armed pragmatic RCT nested in a cohort study (Figure 1). The intervention is redression helmet therapy; the control condition is no helmet therapy (allowing natural recovery). The study starts as a cohort study for children aged two to four months with a positional preference and/or skull deformation (T0). At five months of age (T5), follow-up assessments are performed and parents of children with a moderate to severe skull deformation are invited to participate in the RCT. Eligible children whose parents do not wish to enrol in the RCT are invited to join the non-randomised controlled trial (nRCT) that runs parallel to the RCT. In both studies, follow-up assessments are performed at eight (T8), twelve (T12) and twenty-four months (T24) of age.

Ethics approval for the study was given on the 8th January 2009 (ref: NL24352.044.08) by the Medical Ethics Committee of the Medisch Spectrum Twente hospital in Enschede, The Netherlands.

### Recruitment & setting

Participants were recruited (April 2009 to present) and measured by specially trained paediatric physical therapists (HEADS PPTs) in the Eastern part of the Netherlands (in the provinces of Drenthe, Overijssel and parts of Gelderland).

In The Netherlands, all infants are screened in the first months of life for positional preference and skull deformation at well-baby clinics. Youth Health Care professionals working at well-baby clinics in the region where the study is carried out have been informed about this study, reminded to look for this condition and asked to refer cases to HEADS PPTs.

There are 96 HEADS PPTs involved in the study, working in 73 physical therapist practices. They all received three instruction sessions from the researchers of the HEADS study, including theory lessons on positional preference and skull deformation, a refresher course about plagiocephalometry (PCM) assessment and training in recruiting patients for RCTs.

Based on their experience and performance in the HEADS study, six HEADS PPTs were selected to perform the assessments at T24 (T24-HEADS PPTs) and received an extra instruction session.

Children could be treated with helmet therapy at ProReva (Zwolle), Deventer Hospital/LIVIT (Deventer) and Slingeland Hospital/Roessingh Rehabilitation Technique (Doetinchem). At the start of the HEADS study, these were the only institutions providing helmet therapy within the region in which the project is carried out, and therefore they were asked to collaborate in the RCT. Parents of children in the nRCT could also choose institutions outside of this region or newer institutes that provide helmet therapy within the region.

### Eligibility criteria

#### *Cohort study*

Children aged two to four months with a positional preference and/or skull deformation are eligible for the cohort study. Premature children (gestational age below 36 weeks), children with congenital muscular torticollis, craniosynostosis and/or dysmorphic features are all excluded.

#### *Randomised controlled trial*

Children aged 5 months with a moderate to severe skull deformation, measured by PCM are eligible for the RCT. PCM is a reliable, valid, non-invasive and easy-to-use method for measuring the shape of the skull [35,36]. To determine the severity of deformational plagiocephaly, the oblique diameter difference index (ODDI) is used. This is the ratio between the longest and the shortest oblique diameter, multiplied by 100%. Both diameters are located at 40° from the anterior-posterior line. A moderate to severe plagiocephaly is defined as 108% ≤ ODDI ≤ 113%. The severity of deformational brachycephaly is established with the cranio proportional index (CPI). This is the ratio between the width and the length of the skull and is considered to be moderate to severe when 95% ≤ CPI ≤ 104%. Mixed forms with ODDI > 106% and CPI > 92% are also included. Exclusion criteria are similar to those at T0.

At T5, children meeting RCT eligibility criteria can still enrol in the study (late-enrolment).

#### *Non-randomised controlled trial*

Children eligible for the RCT, but whose parents declined participation, are invited to participate in the nRCT for follow-up. Children with PCM outcomes above the upper thresholds of the inclusion criteria for the RCT are also asked to participate in the nRCT.

### Population

Figure 1 shows that 883 infants enrolled at T0 for baseline measurement. At T5, 808 infants had a follow-up assessment; 477 did not meet the inclusion criteria for the RCT. Of these 477 infants, 26 infants had PCM outcomes above the upper thresholds of the inclusion criteria for the RCT and were eligible to participate in the nRCT. Seventy-five infants enrolled at T5 via late-enrolment, of whom 5 infants had PCM outcomes above the upper thresholds of the inclusion criteria for the RCT and were eligible to participate in the nRCT. Of the eligible 401 infants, 84 (21%) were recruited for the RCT, 296 did not participate in the RCT because their parents declined to enrol them, but were recruited for the nRCT (74%) and 21 (5%) were not recruited to either of the studies. Parents signed an informed consent form before participation in the cohort study, as well as before participation in the RCT.

### Randomisation

A computer-generated blocked randomisation plan with blocks of eight participants is used to allocate treatment in the RCT. After a HEADS PPT enrols a child for the RCT, he or she informs the researcher (RMW) who contacts the parents. Both parents and researcher are unaware of allocation until the parents have signed the informed consent form and confirmed participation. The researcher performs the allocation and informs the parents about group allocation. The child’s HEADS PPT, general practitioner and Youth Health Care professional are also informed about the allocation afterwards.

### Blinding

Blinding of parents and professionals to allocation is not possible during the intervention period, including the T8 and T12 assessment. To ensure unbiased long-term outcomes, the T24 assessments are blinded. These assessments are carried out by T24-HEADS PPTs, who are unfamiliar with the history of the infants they are measuring. Furthermore, we instruct parents in the invitation letter and a poster at the assessment location, not to mention group allocation to the assessor.

### Interventions

#### *Randomised controlled trial*

Helmet therapy: parents of participants allocated to the helmet therapy group were asked to make an appointment at one of the three collaborating institutes for helmet therapy. First a (paediatric) physician was consulted to confirm diagnosis and exclude contraindications. Subsequently, the orthotist provided care as usual; he constructed the custom-made helmet, supplied information about introducing the helmet to the infant, regular wearing instructions and instructions about cleaning of the helmet and general care. The helmet has to be worn for at least 23 hours per day from six to twelve months of age.

No helmet therapy: Parents of participants allocated to the no helmet therapy group were asked not to start any treatment for the skull deformation of their child. In this group, recovery of deformation of the head was awaited by allowing spontaneous growth of the skull.

#### *Non-randomised controlled trial*

In the nRCT, parents were able to select a treatment for their child, that is, either helmet therapy or no helmet therapy. The choice was recorded afterwards when the child was twelve months old (T12).

### Data collection

The cohort study started with a baseline measurement at two to four months of age (T0). A follow-up measurement was performed in all children at 5 months of age (T5).

In the RCT, assessments took place at the age of 8 months (T8), 12 months (T12) and 24 months (T24) (Figure 1). In the nRCT the same assessments took place at T12 and T24. At T8 only a parental questionnaire was collected by mail.

Data were collected by the HEADS PPTs. During every assessment, the shape of the skull was measured, a motor assessment was carried out and both the parents and the HEADS PPTs were asked to complete a questionnaire. The HEADS PPT sent the data about each child to the researcher (RMW).

#### *Baseline characteristics*

Through the parental questionnaire at T0 and the parental questionnaire for late-enrolment at T5, information about background characteristics, medical characteristics and other possible prognostic factors were collected.

#### *Primary outcome*

The primary outcome is the transverse shape of the skull at 24 months, measured with PCM. The severity of deformational plagiocephaly was determined using the ODDI, and ear deviation (ED) was calculated to determine ear misalignment. The severity of deformational brachycephaly was determined by the CPI. A continuous outcome variable (change in score from pre- to post-test) as well as a dichotomous outcome variable will be used for analysis. The dichotomous variable distinguishes full recovery from no full recovery with a cut-off for full recovery of ODDI < 104% and CPI < 90%.

#### *Secondary outcomes*

Secondary outcomes are 1) satisfaction of the parents and HEADS PPT with skull shape, face and body (5-point Likert scale); 2) psychomotor development (a modified Gesell assessment, at regular well-baby clinic visits) [37]; 3) motor domain of Bayley Scales of Infant Development (BSID III) [38]; 4) anxiety level of parents (Spielberger State-Trait Anxiety Inventory, Dutch version) [39]; 5) parental concerns about the child’s future, possible teasing and uncertainty about the child’s appearance (5-point Likert scale); 6) quality of life (Infant Toddler Quality of Life Questionnaire (ITQOL-SF47) [40]) and 7) parental satisfaction with treatment.

#### *Compliance*

The questionnaire at T12 assessed whether parents were compliant with the therapy to which their child was assigned. Also recorded, was whether parents switched groups, and if they did, the age this happened and the reason for it. The helmet providers also collected start and end dates of helmet therapy given to infants in the RCT. Furthermore, helmets in the RCT of the HEADS study are equipped with a logging device (LoD). The LoD measures the number of hours a helmet is worn per week (therapy compliance) and will be used to determine a dose–response relationship. The LoD was attached to the helmet and data were sent to the researcher after the invention period.

In both groups, parents were asked at T12 whether they provided extra care to treat the skull deformation of their child, such as the use of positioning devices, performing exercises with their child or applying various additional therapies.

#### *Determination of costs*

Cost data were collected alongside the effectiveness study. Both medical costs and indirect costs incurred by parents because of diagnostic work-up and treatment were recorded. Indirect costs were collected with the help of a diary completed by parents during the intervention period. Costs are being determined for both the RCT and the nRCT.

### Sample size

The required sample size for the HEADS RCT, based on a significance level of 5%, power of 90% and a difference in mean improvement of at least 4 ODDI-points (SD 6 ODDI-points) was calculated as 72 infants (36 in each arm). Assuming a maximum estimated loss-to-follow up of 25%, we needed to include 96 children in the RCT.

In 2008, a preliminary study was performed into the feasibility of an RCT on helmet therapy for skull deformation. Of the parents of 61 children with a skull deformation, 39% agreed to participate in a study as described in the patient information and verbally clarified. In the light of this information, the size of the current study region was chosen and the inclusion period was estimated.

### Statistical analyses

Data analyses will be performed using SPSS 18.0. A statistical significance level of 0.05 will be used and missing values will be imputed with multiple imputation [41].

#### *Cohort study*

Data analysis will start with descriptive statistics of baseline demographic and clinical characteristics of the total population at T0. At T5, this will be repeated for the clinical characteristics.

#### *Randomised controlled trial*

At T5, characteristics of the RCT population will be described. In a subsequent analysis, the intervention and control group will be compared with respect to prognostic factors using the independent samples *t*-test or the chi square test. The representativeness of the RCT population will be determined by comparing baseline demographic and clinical characteristics of the RCT population with those of the total eligible population at T5. Both the change score (continuous variable) and the success of recovery (dichotomous variable) will be compared between groups on an intention-to-treat basis. After analysis of covariance (ANCOVA), both multiple regression analyses (change score) and logistic regression analyses (success of recovery) will be carried out with predictor variables to control for confounders. Finally, a per-protocol analysis will be performed.

#### *Non-randomised controlled trial*

Baseline characteristics and applied therapies will be described for participants in the nRCT and compared between children treated with a helmet and children whose parents chose not to start helmet therapy. Similarly to the RCT, both the continuous and the dichotomous variables will be compared between groups on an intention-to-treat basis. After univariate analyses, both multivariate and logistic regression analyses will be carried out adding predictor variables.

#### *Comparison between the randomized and the non-randomised controlled trials*

Baseline characteristics will be compared between the RCT and the nRCT. To study differences in the continuous as well as the dichotomous variable between the RCT and the nRCT, both a multiple linear regression analysis and a logistic regression analysis will be carried out, with the interaction factor of study (RCT or nRCT) × group (helmet or no helmet).

## Discussion

The HEADS trial is the first study to present an RCT on the long-term effects of helmet therapy compared to no helmet therapy in infants with moderate to severe skull deformation. The HEADS study started as a cohort study for infants aged two to four months, and continued as an RCT after the first follow-up assessment at the age of five months. In parallel with the RCT, a non-randomised controlled trial (nRCT) was carried out. This extensive cohort study will provide excellent opportunities to study the determinants of skull deformation. Outcomes of the RCT and the nRCT will provide objective information about treatment options for the parents of affected children. With this information, an informed decision can be made whether to start helmet therapy or not. Additionally, outcomes from the cost-effectiveness study are expected to influence future treatment and reimbursement policies.

Recruitment in RCTs is often a challenge and it is common that trials fail to reach their target sample size [42]. In the RCT of the HEADS study, enrolment also proved more difficult than expected. During the recruitment period, it gradually became clear that only 21% of the parents of eligible infants gave consent for the RCT (Figure 1). This is half of the 39% enrolment rate predicted in the preliminary study, and questions the validity of a preliminary study. A much longer recruitment period is needed to recruit the calculated sample size of 96, necessary in case of a maximal loss to follow-up of 25%.

However, most parents refusing participation in the RCT are willing to enrol in the nRCT. Figure 1 shows that only 21 participants who were eligible for participation in the RCT or nRCT were not recruited, implying that almost the complete group of eligible patients at T5 (n = 331) from the original cohort was followed in the HEADS study. This emphasizes the advantage of the nested RCT design; due to their participation in the cohort study, participants are already committed to the study once the RCT and nRCT recruitment starts.

Another methodological advantage of the present study design is its ability to better evaluate the representativeness of the RCT study population. Usually, RCTs have homogeneous yet very selective populations to maximize the likelihood of detecting significant differences. The cohort study of the HEADS study represents a broad population. Due to the nested study design, it is possible to determine the external validity of the RCT, by testing whether the RCT population is representative of the broad, eligible population at T5. The same can be determined for the nRCT population. Furthermore, we can study whether participants in the RCT are comparable to the nRCT participants by comparing the study outcomes and baseline characteristics in both studies.

Finally, as the decision for helmet therapy in the nRCT group was made by parents themselves, this will allow us to investigate the relationship between real-world decisions and treatment outcomes. This provides more information on the usefulness of data from non-randomised compared to randomised studies, which is relevant in comparative effectiveness research. Furthermore simultaneous analysis of data from an RCT and nRCT can strongly contribute to the generalizability of the study outcomes and the development of clinical practice guidelines as compared to single RCTs [43].

Final results of the HEADS study are expected in 2013.

### Trial status

The trial is ongoing.

## Abbreviations

ANCOVA, analysis of covariance; BSID, Bayley Scales of Infant Development; CPI, cranio proportional index; ED, ear deviation; ITQOL-SF47, Infant Toddler Quality of Life Questionnaire; LoD, logging device; nRCT, non-randomised controlled trial; ODDI, oblique diameter difference index; PCM, plagiocephalometry; PPT, paediatric physical therapist; RCT, randomized controlled trial.

## Competing interests

The authors declare that they have no competing interests.

## Authors’ contributions

RMW is responsible for the day-to-day management of the HEADS study, collected the data and wrote the main article. MMB and LAV designed the study and were responsible for the grant application. MMB is the principal grant holder and has overall responsibility for the conduct of the trial. CGMC contributed to both the design of study and the grant application with respect to the statistical analyses. MJI contributed to the design of the study, grant application and writing the article. All authors read and approved the final manuscript.

## References

[B1] Boere-Boonekamp MM van der Linden-Kuiper LT Positional preference: prevalence in infants and follow-up after two years Pediatrics 2001 107 339 343 10.1542/peds.107.2.339 11158467

[B2] Mulliken JB Vander Woude DL Hansen M LaBrie RA Scott RM Analysis of posterior plagiocephaly: deformational versus synostotic Plast Reconstr Surg 1999 103 371 380 10.1097/00006534-199902000-00003 9950521

[B3] Argenta L David L Thompson J Clinical classification of positional plagiocephaly J Craniofac Surg 2004 15 368 372 10.1097/00001665-200405000-00004 15111792

[B4] Clarren SK Plagiocephaly and torticollis: etiology, natural history, and helmet treatment J Pediatr 1981 98 92 95 10.1016/S0022-3476(81)80549-5 7452415

[B5] Collett B Breiger D King D Cunningham M Speltz M Neurodevelopmental implications of "deformational" plagiocephaly J Dev Behav Pediatr 2005 26 379 389 10.1097/00004703-200510000-00008 16222180 PMC3393045

[B6] Balan P Kushnerenko E Sahlin P Huotilainen M Naatanen R Hukki J Auditory ERPs reveal brain dysfunction in infants with plagiocephaly J Craniofac Surg 2002 13 520 525 discussion 526 10.1097/00001665-200207000-00008 12140415

[B7] Miller RI Clarren SK Long-term developmental outcomes in patients with deformational plagiocephaly Pediatrics 2000 105 E26 10.1542/peds.105.2.e26 10654986

[B8] Hutchison BL Hutchison LA Thompson JM Mitchell EA Plagiocephaly and brachycephaly in the first two years of life: a prospective cohort study Pediatrics 2004 114 970 980 10.1542/peds.2003-0668-F 15466093

[B9] Hutchison BL Stewart AW Mitchell EA Deformational plagiocephaly: a follow-up of head shape, parental concern and neurodevelopment at ages 3 and 4 years Arch Dis Child 2010 96 85 90 20880942 10.1136/adc.2010.190934

[B10] van Vlimmeren LA van der Graaf Y Boere-Boonekamp MM L'Hoir MP Helders PJ Engelbert RH Effect of pediatric physical therapy on deformational plagiocephaly in children with positional preference: a randomised controlled trial Arch Pediatr Adolesc Med 2008 162 712 718 10.1001/archpedi.162.8.712 18678802

[B11] Bialocerkowski AE Vladusic SL Wei Ng C Prevalence, risk factors, and natural history of positional plagiocephaly: a systematic review Dev Med Child Neurol 2008 50 577 586 10.1111/j.1469-8749.2008.03029.x 18754894

[B12] Hutchison BL Stewart AW Mitchell EA Characteristics, head shape measurements and developmental delay in 287 consecutive infants attending a plagiocephaly clinic Acta Paediatr 2009 98 1494 1499 10.1111/j.1651-2227.2009.01356.x 19548915

[B13] Hutchison BL Thompson JM Mitchell EA Determinants of nonsynostotic plagiocephaly: a case–control study Pediatrics 2003 112 e316 10.1542/peds.112.4.e316 14523218

[B14] van Vlimmeren LA van der Graaf Y Boere-Boonekamp MM L'Hoir MP Helders PJ Engelbert RH Risk factors for deformational plagiocephaly at birth and at 7 weeks of age: a prospective cohort study Pediatrics 2007 119 e408 e418 10.1542/peds.2006-2012 17272603

[B15] McKinney CM Cunningham ML Holt VL Leroux B Starr JR A case–control study of infant, maternal and perinatal characteristics associated with deformational plagiocephaly Paediatr Perinat Epidemiol 2009 23 332 345 10.1111/j.1365-3016.2009.01038.x 19523080

[B16] Michels AC van den Elzen ME Vles JS van der Hulst RR Prevalence of positional plagiocephaly and excessive folic acid intake during pregnancy Cleft Palate Craniofac J 2012 49 1 4 10.1597/09-096 21740174

[B17] Weernink MGM van Wijk RM Groothuis-Oudshoorn CGM Van Vlimmeren LA Lanting CI Boere-Boonekamp MM De relatie tussen vitamine-D-inname en schedeldeformatie bij zuigelingen [abstract] Tijdschrift voor Jeugdgezondheidszorg 2011 4 1

[B18] Hutchison BL Stewart AW De Chalain TB Mitchell EA A randomised controlled trial of positioning treatments in infants with positional head shape deformities Acta Paediatr 2010 99 1556 1560 10.1111/j.1651-2227.2010.01872.x 20491708

[B19] Pollack IF Losken HW Fasick P Diagnosis and management of posterior plagiocephaly Pediatrics 1997 99 180 185 10.1542/peds.99.2.180 9024443

[B20] Lipira AB Gordon S Darvann TA Hermann NV Van Pelt AE Naidoo SD Govier D Kane AA Helmet versus active repositioning for plagiocephaly: a three-dimensional analysis Pediatrics 2010 126 e936 e945 10.1542/peds.2009-1249 20837585

[B21] Littlefield TR Cranial remodeling devices: treatment of deformational plagiocephaly and postsurgical applications Semin Pediatr Neurol 2004 11 268 277 10.1016/j.spen.2004.10.004 15828711

[B22] Loveday BP de Chalain TB Active counterpositioning or orthotic device to treat positional plagiocephaly? J Craniofac Surg 2001 12 308 313 10.1097/00001665-200107000-00003 11482615

[B23] Vles JS Colla C Weber JW Beuls E Wilmink J Kingma H Helmet versus nonhelmet treatment in nonsynostotic positional posterior plagiocephaly J Craniofac Surg 2000 11 572 574 10.1097/00001665-200011060-00010 11314498

[B24] Lee A Van Pelt AE Kane AA Pilgram TK Govier DP Woo AS Smyth MD Comparison of perceptions and treatment practices between neurosurgeons and plastic surgeons for infants with deformational plagiocephaly Journal of Neurosurgery: Pediatrics 2010 5 368 374 10.3171/2009.11.PEDS0983 20367342

[B25] Thorpe KE Zwarenstein M Oxman AD Treweek S Furberg CD Altman DG Tunis S Bergel E Harvey I Magid DJ Chalkidou K A pragmatic-explanatory continuum indicator summary (PRECIS): a tool to help trial designers J Clin Epidemiol 2009 62 464 475 10.1016/j.jclinepi.2008.12.011 19348971

[B26] Macpherson H Pragmatic clinical trials Complement Ther Med 2004 12 136 140 10.1016/j.ctim.2004.07.043 15561524

[B27] Schwartz D Lellouch J Explanatory and pragmatic attitudes in therapeutical trials J Chronic Dis 1967 20 637 648 10.1016/0021-9681(67)90041-0 4860352

[B28] Fransen GAJ van Marrewijk CJ Mujakovic S Muris JWM Laheij RJF Numans ME de Wit NJ Samsom M Jansen JBMJ Knottnerus JA Pragmatic trials in primary care Methodological challenges and solutions demonstrated by the DIAMOND-study. BMC Med Res Methodol 2007 7 16 10.1186/1471-2288-7-16 PMC1865384 17451599

[B29] Tunis SR Stryer DB Clancy CM Practical clinical trials: increasing the value of clinical research for decision making in clinical and health policy JAMA 2003 290 1624 1632 10.1001/jama.290.12.1624 14506122

[B30] Relton C Torgerson D O'Cathain A Nicholl J Rethinking pragmatic randomised controlled trials: introducing the "cohort multiple randomised controlled trial" design BMJ 2010 340 c1066 c1066 10.1136/bmj.c1066 20304934

[B31] Donovan J Mills N Smith M Brindle L Jacoby A Peters T Frankel S Neal D Hamdy F Quality improvement report: Improving design and conduct of randomised trials by embedding them in qualitative research: ProtecT (prostate testing for cancer and treatment) study Commentary: presenting unbiased information to patients can be difficult. BMJ 2002 325 766 770 10.1136/bmj.325.7367.766 PMC1124277 12364308

[B32] Ross S Grant A Counsell C Gillespie W Russell I Prescott R Barriers to participation in randomised controlled trials: a systematic review J Clin Epidemiol 1999 52 1143 1156 10.1016/S0895-4356(99)00141-9 10580777

[B33] Bartlett C Doyal L Ebrahim S Davey P Bachmann M Egger M Dieppe P The causes and effects of socio-demographic exclusions from clinical trials Health Technol Assess 2005 9 1 152 iii-iv, ix-x 10.3310/hta9380 16181564

[B34] Bonell C Oakley A Hargreaves J Strange V Rees R Assessment of generalisability in trials of health interventions: suggested framework and systematic review BMJ 2006 333 346 349 10.1136/bmj.333.7563.346 16902217 PMC1539056

[B35] van Adrichem LNA van Vlimmeren LA Cadanova D Helders PJM Engelbert RHH van Neck HJW Koning AHJ Validation of a simple method for measuring cranial deformities (Plagiocephalometry) J Craniofac Surg 2008 19 15 21 18216659 10.1097/scs0b013e31815c93cb

[B36] van Vlimmeren LA Takken T van Adrichem LN van der Graaf Y Helders PJ Engelbert RH Plagiocephalometry: a non-invasive method to quantify asymmetry of the skull; a reliability study Eur J Pediatr 2006 165 149 157 10.1007/s00431-005-0011-1 16211401

[B37] Laurent Angulo MS, Brouwers-de Jong EA, Bijlsma-Schlösser JFM, Bulk-Bunschoten AMW, Pauwels JH, Steinbuch-Linstra I Ontwikkelingsonderzoek in de jeugdgezondheidszorg. Het Van Wiechenonderzoek – De Baecke-Fassaert Motoriektest 2005, Assen. Van Gorcum

[B38] Bayley N Manual for the Bayley Scales of Infant and Toddler Development 3rd edition 2006 Psychological Corp, San Antonio, TX

[B39] van der Ploeg HM Defares PB Spielberger CD Handleiding bij de Zelf-Beoordelings Vragenlijst, ZBV: Een Nederlandstalige bewerking van de Spielberger State-Trait Anxiety Inventory 1980 Swets & Zeitlinger, Lisse

[B40] Raat H Landgraf JM Oostenbrink R Moll HA Essink-Bot M-L Reliability and validity of the Infant and Toddler Quality of Life Questionnaire (ITQOL) in a general population and respiratory disease sample Qual Life Res 2006 16 445 460 17111231 10.1007/s11136-006-9134-8 PMC2792359

[B41] Van Buuren S Groothuis-Oudshoorn CGM mice: Multivariate Imputation by Chained Equations in R J Stat Softw 2011 45 1 67

[B42] Haidich AB Ioannidis JPA Patterns of patient enrollment in randomised controlled trials J Clin Epidemiol 2001 54 877 883 10.1016/S0895-4356(01)00353-5 11520646

[B43] Petticrew M Roberts H Evidence, hierarchies, and typologies: horses for courses J Epidemiol Community Health 2003 57 527 529 10.1136/jech.57.7.527 12821702 PMC1732497

